# Crosstalk between microRNA and oxidative stress in ovarian cancer: diagnosis, pathogenesis and therapeutic resistance

**DOI:** 10.1007/s12032-025-03024-5

**Published:** 2025-12-27

**Authors:** Amany Gomaa Atiaa, Shehab M. Abd E-Kader, Doha El-Sayed Ellakwa

**Affiliations:** 1 Department of Surgery, Faculty of Physical Therapy, Sinai University, Kantra Branch, Ismailia, Egypt; 2 Department of Physical Therapy, Faculty of Medical Rehabilitation Sciences, King Abdulaziz University, Jeddah, Saudi Arabia; 3 Department of Cardiopulmonary Disorders and Geriatrics, Faculty of Physical Therapy, Cairo University, Cairo, Egypt; 4 Department of Biochemistry and Molecular Biology, Faculty of Pharmacy for Girls, Al-Azhar University, Cairo, Egypt; 5 Department of Biochemistry, Faculty of Pharmacy, Sinai University, Kantra Branch, Ismailia, Egypt

**Keywords:** Ovarian cancer, Oxidative stress, Reactive oxygen species, MicroRNAs, Redox signaling, Chemoresistance, Biomarkers, Nanotherapy

## Abstract

Ovarian cancer (OC) is the most lethal gynecologic malignancy due to late-stage diagnosis, frequent recurrence, and resistance to therapy. Emerging evidence highlights oxidative stress (OS)—a redox imbalance caused by excessive reactive oxygen species (ROS)—as a key contributor to tumor development and therapy failure. This article presents a narrative review of the bidirectional relationship between oxidative stress and microRNAs (miRNAs) in OC, emphasizing their molecular crosstalk, clinical relevance, and therapeutic potential. A targeted synthesis of recent experimental and clinical studies was conducted to explore how redox biology and miRNA dysregulation contribute to OC pathogenesis and treatment resistance. ROS promotes genomic instability, epithelial–mesenchymal transition (EMT), angiogenesis, immune evasion, and chemoresistance. Redox-responsive miRNAs (e.g., miR-29b, miR-200a/c, miR-145-5p, miR-484, miR-21) regulate antioxidant defenses, DNA repair, apoptosis. OS modulates miRNA biogenesis via transcriptional and epigenetic changes, and miRNAs form feedback loops that influence ROS levels and tumor progression. Circulating and exosomal miRNAs show promise as non-invasive biomarkers, but require further clinical validation. Therapeutic approaches targeting the ROS–miRNA axis—including mimics, antagomiRs, and nanocarriers—show preclinical potential, though challenges in delivery and toxicity remain. The dynamic OS–miRNA interplay represents a novel regulatory axis in OC. Exploiting this axis may enhance early diagnosis and therapy. Future work should integrate redox profiling with miRNA expression to personalize treatment and assess performance relative to existing modalities like PARP inhibitors.

## Introduction

Ovarian cancer (OC) remains the most lethal form of gynaecological cancer globally, a mortality rate largely attributable to late-stage diagnosis, frequent relapses, and the evolution of resistance to standard platinum-based therapies [1], [2]. Among the most significant drivers of OC advancement is oxidative stress (OS), perceived as an excess of reactive oxygen species (ROS) relative to the cell’s antioxidant capacity [3]. ROS serve vital roles in transducing physiological signals,however, unchecked accumulation provokes selective damage to nucleic acids, proteins, and membrane lipids, in turn fuelling genomic instability, epithelial-to-mesenchymal transition (EMT), angiogenic sprouting, and diminished sensitivity to chemotherapy [4]. Intriguingly, augmented ROS concentrations can simultaneously trigger apoptotic pathways and foster resistance to cytotoxic agents, underscoring a bifunctional and microenvironmentally contingent redox milieu in OC [5]. As a salient illustration, Nrf2 pathway upregulation enhances the cell’s antioxidant arsenal yet also correlates with a diminished response to chemotherapy [6]. MicroRNAs (miRNAs) are small non-coding RNAs involved in post-transcriptional gene regulation. Their dysregulation in OC *has been shown to affect* cell proliferation, metastasis, and treatment response [7]. ROS modulates miRNA biogenesis via epigenetic changes, while some miRNAs, in turn, regulate ROS production, forming complex redox-sensitive feedback loops that influence tumour progression [8]. *Key examples include* the miR-200 family (which regulates EMT), miR-29b (targets DNA repair via SIRT1), miR-484 (influences mitochondrial function), and miR-21 (promotes PI3K/AKT signalling) [9]. Their presence in serum, exosomes, and tumour tissues makes miRNAs attractive *candidates* for diagnostic, prognostic, and therapeutic monitoring purposes [10]. However, *inter-patient variability and the absence of standardized protocols for normalization and quantification* remain major barriers to clinical translation [11].Therapeutically, *emerging strategies aim to restore or inhibit specific miRNAs using synthetic mimics or inhibitors (antagomiRs)* to re-establish tumor suppressor function or attenuate oncogenic pathways. *AntagomiRs* are synthetically designed molecules are used to neutralize microRNA function in cells for desired responses [12]. Advances in nanotechnology offer promise for improved targeted delivery, although challenges related to *bioavailability, immunogenicity, and pharmacokinetics* persist [13].

## The role of oxidative stress in ovarian cancer

Ovarian cancer (OC) continues to rank as the most fatal gynecologic cancer primarily because most cases are diagnosed at an advanced stage, accompanied by substantial recurrence rates and enduring resistance to platinum-based chemotherapeutics [14]. Oxidative stress (OS), defined as the disturbance of redox equilibrium that favors reactive oxygen species (ROS) over the antioxidant defense system, has emerged as a critical driver of these clinical aggressiveness features [12]. Elevated levels of OS biomarkers, including nitric oxide (NO) and malondialdehyde (MDA), correlate with later-stage disease and unfavorable outcomes in patients with OC [15]. Although ROS are intrinsic byproducts of fundamental energy pathways, including mitochondrial oxidative phosphorylation and NADPH oxidase activity, their pathological overproduction in OC cells is augmented by metabolic rewiring and the oxidative challenge posed by genotoxic interventions such as chemotherapy and radiotherapy [3], [16]. Mitochondrial impairment serves as a predominant origin of reactive oxygen species in ovarian cancer cells, with superoxide production primarily arising from localized electron transport chain “leaks” [17]. Concurrently, the nicotinamide adenine dinucleotide phosphate oxidase family—most notably NOX4 and NOX5—further contributes to ROS buildup, triggered by pro-tumor and inflammatory signals [18]. In addition, the hypoxic microenvironment characteristic of the ovarian cancer tumor microenvironment destabilizes oxidative phosphorylation and exacerbates ROS generation [19].

### Impact of ROS on tumor biology

**Fig. 1:**
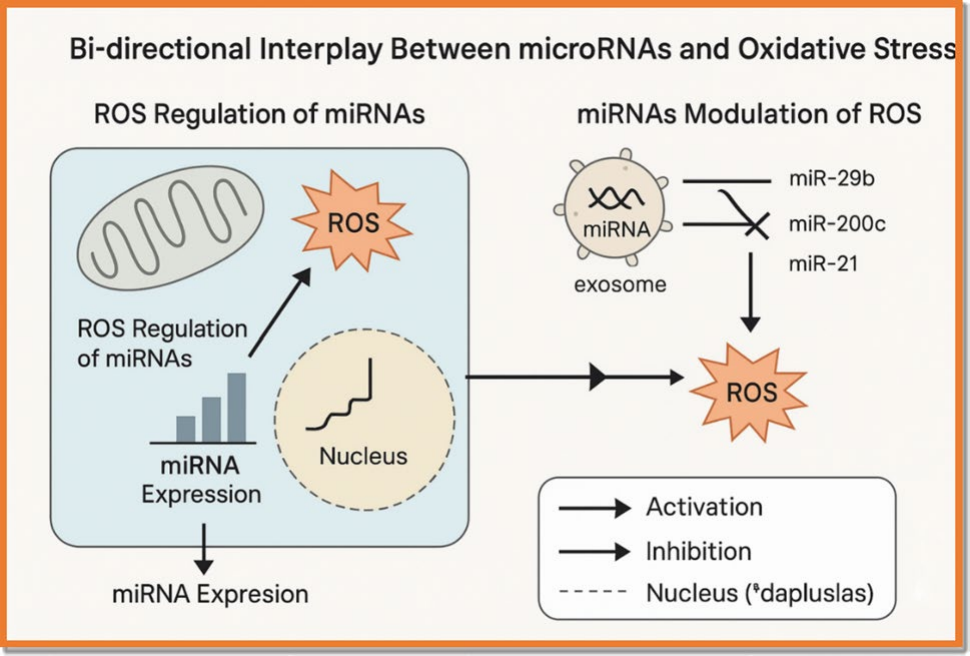
Bi-directional Interplay Between microRNAs and Oxidative Stress. This diagram illustrates the reciprocal regulatory relationship between reactive oxygen species (ROS) and microRNAs (miRNAs) in ovarian cancer (OC). ROS modulate miRNA expression through transcription factors (e.g., p53, NF-κB), while specific miRNAs (e.g., miR-21, miR-200a, miR-29b) regulate oxidative stress responses by targeting antioxidant enzymes, mitochondrial integrity, and signaling cascades such as PI3K/AKT and MAPK

Reactive oxygen species (ROS) induce oxidative modifications in DNA, lipids, and proteins, leading to compromised membrane integrity and disruption of intracellular signaling pathways [20]. These molecular perturbations promote neoplastic growth, foster genomic instability, and contribute to resistance against apoptosis and therapeutic interventions in ovarian cancer [17]. Elevated ROS levels in ovarian carcinoma further stimulate angiogenesis, drive metabolic reprogramming, and facilitate epithelial-to-mesenchymal transition (EMT), a critical process for tumor invasion and metastasis [21]. ROS influence EMT by activating key transcriptional regulators and inducing epigenetic changes, including alterations in DNA methylation patterns [22]. The interplay between ROS and these cellular processes underscores their central role in ovarian cancer pathogenesis and highlights the importance of targeting oxidative stress pathways for improved diagnostic and therapeutic strategies [23].The reciprocal crosstalk between ROS and microRNAs in these processes is schematically depicted in Fig. 1.

### ROS in therapy resistance

Reactive oxygen species (ROS) perform opposing functions in oncologic pharmacotherapy.Agents like cisplatin exploit oxidative DNA lesions to trigger apoptosis; however, excessive ROS can concurrently activate survival pathways, culminating in therapeutic resistance [24]. Ovarian cancer (OC) cells respond to oxidative stress by upregulating their antioxidant machinery, notably glutathione peroxidase and superoxide dismutase (SOD), thereby fortifying their survival under cytotoxic stress [25], [20]). Concurrent activation of transcription factors, primarily nuclear factor-κB (NF-κB) and nuclear factor-erythroid-2-related factor 2 (NRF2), elicits transcriptional programs that promote the expression of anti-apoptotic proteins, enhance drug efflux, and preserve cellular integrity [26, 27]). NRF2 plays a central role in platinum resistance by orchestrating drug biotransformation, potentiating efflux pumps, and maintaining redox equilibrium [6]. Its aberrant hyperactivation not only accelerates the stem-like phenotype of tumor-initiating cells but also fosters disease recurrence. Targeted suppression of NRF2—using small molecules like ML385—augmented with selective inhibitors of glutathione peroxidase 4 (GPX4), effectively precipitates ferroptotic cell death by destabilizing the redox milieu. Additionally, accumulation of p62 can *activate the Keap1-NRF2 axis, further exacerbating therapeutic resistance* [28].

### Oxidative stress and immune evasion

Oxidative stress contributes to immune evasion in OC by remodelling the tumour microenvironment (TME) into an immunosuppressive niche. ROS and reactive nitrogen species (RNS) *directly impair antitumor immune function* and promote chronic inflammation [29], [30]. Persistent OS fosters the polarization of tumor-associated macrophages (TAMs) toward an M2-like phenotype, characterized by secretion of IL-10, TGF-β, and expression of PD-L1 and CTLA-4, *leading to the suppression of T-cell activation and promoting immune escape* [31]. ROS also *stimulate the recruitment of immunosuppressive T regulatory (Treg) cells and myeloid-derived suppressor cells (MDSCs)*, further dampening cytotoxic responses [3]. *Of particular interest, OS facilitates the release of exosomes carrying redox-sensitive miRNAs*. These exosomal miRNAs can reprogram recipient immune cells, *promoting M2 polarization and impairing CD8⁺ T cell cytotoxicity* [32]. This emerging exosome–miRNA axis represents a novel immune evasion mechanism and a *potential target for therapeutic intervention*.

## MicroRNAs in relation to ovarian cancer and the regulation of oxidative stress

Ovarian cancer (OC) development is closely linked to microRNA (miRNA) deregulation and its reciprocal engagement with oxidative stress (OS)—the state arising when reactive oxygen species (ROS) overwhelm cellular antioxidant mechanisms [33], [34]. Such disrupted molecular interplay disturbs intracellular redox homeostasis, fosters malignant evolution, and is implicated in acquired resistance to standard treatments. Dissecting these coordinated alterations may reveal redox-sensitive pathways amenable to novel therapeutic intervention and advance the identification of informative biomarkers [35].

**Fig. 2:**
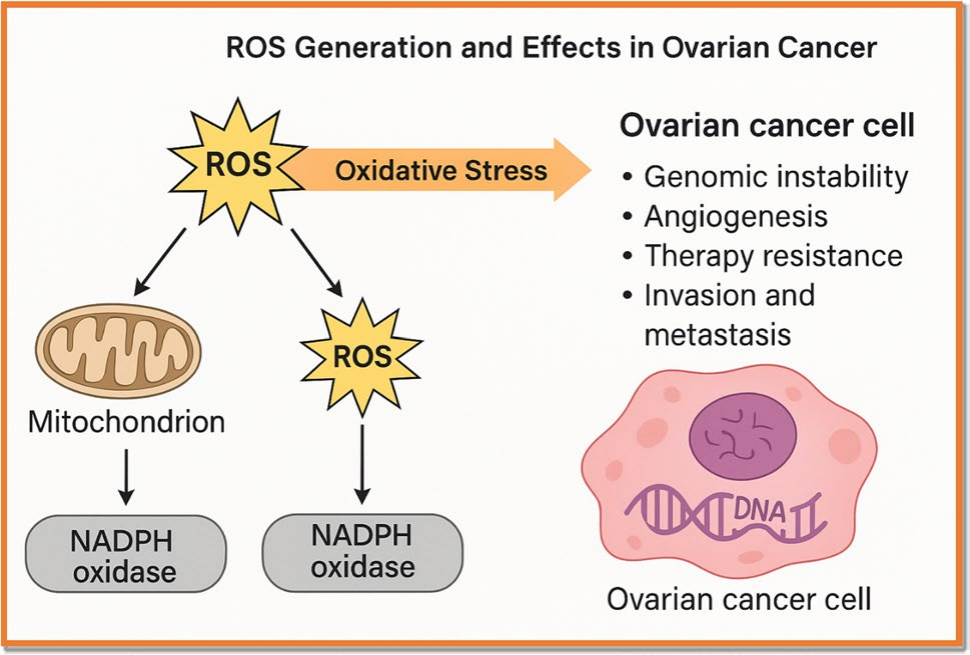
ROS Generation and Effects in Ovarian Cancer. A schematic overview of intracellular and tumor microenvironmental sources of ROS in OC, including mitochondria, NADPH oxidases (NOX), and hypoxia. The figure also shows downstream effects of elevated ROS levels on DNA damage, angiogenesis, immune evasion, and epithelial-to-mesenchymal transition (EMT), contributing to tumor growth and chemoresistance

Figure 2 ROS generation and effects in ovarian cancer.

### Impact of miRNAs on the oxidative stress response

Small non-coding RNAs, particularly miRNAs, exert critical influence over redox balance by fine-tuning intracellular ROS production, orchestrating redox-sensitive signaling cascades, and preserving mitochondrial architecture [36]. MiR-29b, designated a tumor suppressor, inhibits the NAD+ -dependent deacetylase SIRT1, a key regulator of DNA integrity and ROS detoxification. Its loss of expression in ovarian cancer (OC) results in mitochondrial membrane potential collapse, accumulation of oxidative lesions, and accelerated apoptotic signaling [37]. In contrast, enforced SIRT1 expression fosters chemoresistance via augmented antioxidant defenses [38]. Therapies aimed at restoring miR-29b could, therefore, re-engage oxidative stress-induced cytotoxicity and potentiate the efficacy of platinum-based regimens. Components of the miR-200 family, including miR-141 and miR-200a, directly repress MAPK14 (also designated p38α), a redox-sensitive mitogen-activated protein kinase that integrates stress, inflammatory, and DNA damage response signals [39]. Inhibition of this kinase by miR-200 gfamily members attenuates pro-oxidant signaling and DNA repair, underscoring their dual role in mitochondrial protection and preservation of genomic integrity. miR-141-3p promotes M2 macrophage polarization via Keap1-Nrf2 signaling, while miR-200a suppresses *Keratin-19 (KRT19)*, enhancing NRF2-driven antioxidant responses [40]. *MiR-484* represses *SESN2*, a redox-sensitive autophagy mediator, thereby *exacerbating mitochondrial dysfunction, ROS accumulation, and apoptosis* [41, 42]. In contrast, *miR-361-5p* mitigates mitochondrial ROS and preserves mitochondrial health [43].

### Oxidative stress as a regulator of miRNA expression

Oxidative stress is not only regulated by microRNAs (miRNAs) but also modulates their expression and processing, establishing bidirectional feedback loops that critically influence ovarian cancer behavior. Below is a comprehensive table summarizing 20 miRNAs relevant to ovarian cancer, including their targets, functions, and clinical relevance (Table 1).

**Table 1:** Key miRNAs in ovarian cancer: targets, functions and clinical relevance

miRNA	Main Target(s)	Function(s) in Ovarian Cancer	Clinical Relevance	Citations
miR-200a/b/c	ZEB1/2, EMT regulators	Inhibits EMT, metastasis	Biomarker, therapy target	[44, 45]
let-7 family	RAS, HMGA2, cell cycle genes	Suppresses proliferation, migration	Prognostic biomarker	[46]
miR-21	PTEN, integrins	Promotes chemoresistance, proliferation	Linked to cisplatin resistance	[45]
miR-3652	Multiple oncogenes	Regulates proliferation, migration	Diagnostic/prognostic biomarker	[47]
miR-34a	NOTCH1, BCL2, STAT3	Induces apoptosis, inhibits proliferation	Therapeutic target	[48]
miR-214	PTEN, β-catenin	Regulates stemness, chemoresistance	Prognostic marker, therapy target	[45]
miR-199a/b-3p	ZEB1, mTOR, c-Met	Inhibits proliferation, EMT	Prognostic marker, therapy target	[49]
miR-223-3p	SOX11	Promotes proliferation, invasion	Potential therapeutic target	[50]
miR-1827	SPTBN2, BCL2L1	Suppresses proliferation, migration	Prognostic biomarker	[51]
miR-149-3p	TIMP2, CDKN1A	Promotes EMT, chemoresistance	Therapy resistance, biomarker	[52]
miR-205	BCL2, ZEB1, E2F1, TP53	Suppresses tumor growth, EMT	Diagnostic/therapeutic potential	[45]
miR-29b	DNMTs, apoptosis genes	Induces apoptosis, inhibits proliferation	Biomarker, therapy target	[44]
miR-376c	WNT/β-catenin pathway	Regulates stemness, tumor progression	Prognostic marker, therapy target	[53]
miR-651-3p	ZNF703, MEK/ERK pathway	Inhibits EMT, proliferation	Diagnostic/therapeutic potential	[54, 55]

Oxidative stress also activates transcription factors such as *NF-κB, p53, and HIF-1α*, which influence miRNA transcription [56]. ROS can modulate miRNA-processing enzymes, including *Drosha* and *Dicer* [56, 57]. *Acute oxidative stimuli* (e.g., H_2_O_2_) increase miR-29b, whereas *chronic exposure* (e.g., smoking) leads to its downregulation, indicating context-specific miRNA responses [1]. Persistent OS may also repress SESN2 through miR-484, amplifying mitochondrial injury. Age-related *Dicer downregulation* leads to impaired miRNA maturation and contributes to redox imbalance and chemoresistance in OC.

### Clinical implications and therapeutic potential

Deciphering the miRNA–ROS axis may inform redox-responsive therapeutic strategies and precision oncology approaches. Key pathways involved include hypoxia signaling, apoptosis, and the DNA damage response.*MiR-21* and *miR-214* suppress *PTEN*, activating the PI3K/AKT/mTOR pathway, thereby enhancing platinum resistance [58, 59]. *MiR- 497 restoration* reverses these effects and improves therapy sensitivity. Circulating and exosomal miRNAs—including *miR-21-3p, miR-891-5p, miR-320b, and miR-320d*—show potential as biomarkers for recurrence risk and therapy resistance [3] in development to modulate oxidative balance and improve drug response. *ROS-responsive delivery systems* allow localized activation of such therapeutics in the tumor microenvironment [60]. *Emerging technologies*, including *spatial transcriptomics, high-throughput miRNA editing, and redox imaging*, will refine patient stratification and guide miRNA-based combination therapies [61].

## The impact of oxidative stress on microrna expression in ovarian cancer

Oxidative stress (OS), a hallmark of ovarian cancer (OC), disrupts redox balance and contributes to key cancer hallmarks including apoptosis, angiogenesis, epithelial–mesenchymal transition (EMT), therapy resistance, and radiotherapy response [62]. *MicroRNAs (miRNAs), as redox-sensitive post-transcriptional regulators, modulate gene networks that govern OC cell fate and therapeutic outcomes.* This section outlines how oxidative stress and miRNAs interact to influence OC progression [63].

### miRNAs in the regulation of apoptosis and cell cycle

Reactive oxygen species (ROS) initiate apoptosis in OC via DNA damage and downstream signaling cascades. For example, *1C-chalcone derivatives* trigger apoptosis in both cisplatin-sensitive and -resistant OC cells through ROS accumulation and *activation of p21 and NF-κB pathways* [64]. *DCTPP1 knockdown increases ROS and apoptosis, whereas its overexpression promotes cisplatin resistance* [54, 55]. ROS-induced inhibition of the PI3K/AKT pathway also activates mitochondrial apoptosis via *PUMA* [18].

**Fig. 3:**
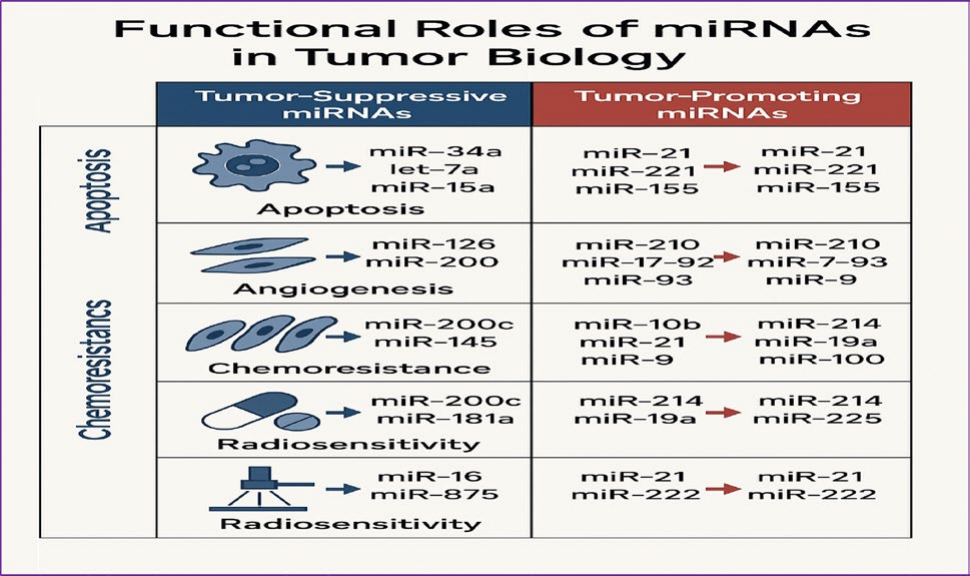
Functional Roles of miRNAs in Tumor Biology. A network-style figure illustrating the multiple roles of miRNAs in regulating apoptosis, angiogenesis, EMT, chemoresistance, radioresistance, and mitochondrial homeostasis in ovarian cancer. Arrows indicate activation or inhibition, with representative miRNAs such as miR-34a, miR-200c, miR-155, and miR-152 annotated at their respective functional nodes

Figure 3 functional roles of miRNAs in tumor biology.

MiRNAs modulate apoptotic balance. The *miR-15/16 cluster* suppresses anti-apoptotic genes such as *BCL2 and Cyclin D1*, sensitizing cells to oxidative injury.*MiR-29b targets SIRT1*, a mitochondrial deacetylase, impairing antioxidant defenses and promoting apoptosis [19]. *Restoring miR-29b may reverse therapy resistance in oxidative-stress-adapted tumors.*

### miRNAs and angiogenesis

Tumor angiogenesis in OC is driven by *hypoxia-mediated ROS*, which activates HIF-1α and VEGF signaling. *MiR-155-5p responds to oxidative stimuli* and suppresses HIF-1α, regulating endothelial differentiation and angiogenesis [12]. *MiR-200b inhibits VEGF-A and ZEB1*, limiting neovascularization [65]. Other angiogenesis-related miRNAs include *miR-204-5p*, which represses *THBS1*, a known angiogenesis inhibitor [66]. *MiR-367 targets LPA1*, reducing vessel formation, while *miR-133a-5p enhances vascularization *via* the TRIM59/VEGF axis* [67]. *MiR-6086 inhibits the OC2/VEGFA/EGFL6 pathway*, making it a potential anti-angiogenic target [68].

### miRNAs in epithelial–mesenchymal transition (EMT)

ROS promote EMT through activation of NF-κB, Snail, and β-catenin signaling. *MiR-200c* reinforces the epithelial phenotype by upregulating *E-cadherin* and suppressing *ZEB1/ZEB2*. *Loss of miR-200c induces mesenchymal traits, metastasis, and chemoresistance* [65]. *ROS downregulate miR-200c*, thus promoting EMT and aggressive tumor behavior [69]. These miRNAs act as redox-controlled switches governing plasticity [70].

### miRNAs and chemoresistance

Redox dysregulation contributes to platinum resistance via antioxidant pathway upregulation, enhanced DNA repair, and altered drug metabolism. *MiR-106b-5p targets OLR1*, and its suppression correlates with cisplatin resistance [71]. *MiR-21 downregulates PTEN*, thereby activating PI3K/AKT signaling and promoting resistance [72]. Though not a redox controller, *miR-21 is ROS-inducible*, creating a feedback loop that amplifies survival signaling. *Targeting miR-21 restores PTEN and enhances treatment efficacy* [68]. Exosomal miRNAs such as *miR-21-3p, miR-891-5p, and miR-320d*, derived from small extracellular vesicles (sEVs), regulate glycolysis, ABC transporters, and DNA repair mechanisms, thus fostering resistance [3]. *MiR-152-3p reverses paclitaxel resistance* by modulating the PTEN/ATG4D-autophagy pathway [71]. *MiR-450b-5p enhances carboplatin resistance* via ACTB and the PI3K/AKT axis [73].

### miRNAs and radioresistance

Although ionizing radiation kills cancer cells through ROS induction, many OC subtypes exhibit radioresistance. *MiR-34a enhances radiosensitivity* by targeting *BCL2 and SIRT1* [74]. *MiR-17-5p suppresses MnSOD*, increasing ROS and sensitizing cells to radiation [28]. MiRNAs such as *miR-200b, miR-141, and miR-1274A* correlate with improved survival post-bevacizumab therapy [58, 59]. Circulating miRNAs like *miR-374a-5p and miR-519d-3p* differentiate therapy responders from non-responders [18]. *Redox-sensitive miRNAs may serve as biomarkers or therapeutic adjuvants to enhance radiosensitivity.*

### miRNAs in mitochondrial dysfunction and ROS accumulation

MiRNAs orchestrate mitochondrial homeostasis and regulate ROS levels. *MiR-361-5p reduces ROS and preserves ATP production*, whereas its inhibition elevates oxidative stress [43]. *MiR-484*, induced by OS, promotes mitochondrial injury and granulosa cell senescence by repressing *SESN2 and YAP1* [41, 52]. These miRNAs also regulate NOX4 and other ROS-generating enzymes, shaping redox signaling [69].

### ROS regulation of miRNA biogenesis

Oxidative stress impacts miRNA biogenesis at transcriptional, post-transcriptional, and epigenetic levels [41]. ROS impairs *Drosha and Dicer*, reducing the generation of mature miRNAs [72]. *Hypoxia and ROS co-suppress Dicer*, as exemplified by *miR-630, which represses Dicer and promotes tumorigenesis* [32]. Reactively generated radicals trigger the activation of key transcriptional regulators—NF-κB, NRF2, and p53—each of which subsequently influences the transcription of miRNA maturation components and their downstream targets.

Configurational epigenetic modifications, including cytosine methylation and specific acetylation/methylation of histones, fine-tune the expression of the microprocessor genes Drosha and Dicer. Dicer depletion induced by oxidative stress promotes a preponderance of onco-miRNAs while concurrently repressing miRNA species with known tumor-suppressive functions [21]. Emergent investigations reveal that m6A RNA methylation further impacts miRNA biogenesis by interacting directly with DGCR8, thereby integrating the effects of redox status into the regulatory circuitry governing miRNA maturation.

## Clinical implications and translational applications of miRNAs in ovarian cancer

Within the field of clinical oncology, microRNAs (miRNAs) linked to oxidative stress have gained attention as potentially valuable biomarkers for diagnosis, prognosis, and therapeutic stratification in ovarian cancer (OC) [75]. The differential expression of these small non-coding RNAs mirrors the intrinsic heterogeneity of the neoplasm, variations in responsiveness to treatment, and the trajectory of disease progression. Their stable presence in biofluids enables their application as minimally invasive biomarkers, facilitating both early detection and longitudinal monitoring of the disease [76].

### Diagnostic and prognostic biomarkers

Ovarian cancer is frequently diagnosed at late stages, emphasizing the need for *early and specific biomarkers*. Circulating miRNAs are attractive candidates due to their *stability, tissue specificity, and resistance to RNase degradation* [72]. For instance, *miR-22 and miR-126 are significantly downregulated in OC serum*, correlating with tumor grade and stage—*with miR-22 outperforming CA-125 in diagnostic accuracy* [77]. Members of the *miR-200 family (miR-141, miR-200a, miR-200c)* are elevated in ascitic fluid and plasma of epithelial OC patients and also regulate *EMT and redox signaling* [78].

*MiR-21 consistently shows high expression across cancers, including OC* (AUC ~ 0.87), and serves as a multi-cancer diagnostic tool [58, 59]. However, *miR-484 requires further validation despite its link to oxidative stress* [79]. In prognostic settings, *low miR-106b-5p and miR-199b-3p levels are associated with platinum resistance and shorter survival*, partly due to their regulation of ROS-related genes such as OLR1. *High miR-21 expression correlates with poor overall survival*. *Exosomal miRNAs like miR-34a, miR-1260a, miR-320d, and miR-4479 differentiate OC patients from healthy controls and track treatment response* [80]. *Combining exosomal miRNAs with CA-125 enhances diagnostic precision for early detection and recurrence monitoring *[81] (see Table 2)*.*

**Table 2:** Clinical applications of redox-associated miRNAs in ovarian cancer

Application	Key miRNAs	Clinical use	Notes
Diagnosis	miR-22, miR-126, miR-141	Liquid biopsy for early detection	↑ Sensitivity/specificity compared to CA-125
Prognosis	miR-106b-5p, miR-3652	Predict recurrence/survival	Involves ROS and inflammation-related gene targets
Chemoresistance	miR-29b, miR-200a, miR-484	Predict platinum response	Linked to redox and apoptosis pathways
Radiosensitivity	miR-34a, miR-17-5p	Predict radiation sensitivity	Modulate oxidative stress response post-irradiation
Therapeutic Targeting	miR-29b (mimic), miR-484 (antagomiR)	Preclinical gene modulation	Phase I validation in progress

### Therapeutic strategies targeting miRNAs

Both miRNA-based approaches and therapies with PARP inhibitors offer innovative options in the treatment of ovarian cancer, working on different molecular pathways of the disease [82]. PARP inhibitors, including olaparib, have emerged as a mainstay in the treatment of ovarian cancers with BRCA mutations and those that are HRD positive, due to their apparent efficacy in advanced disease. However, development of resistance and limited effectiveness in advanced disease remains a critical hurdle [83, 84]. On the other hand, miRNA-based approaches aim to regulate the expression of particular genes that are related to tumor development, metastasis, and resistance to therapies. Some miRNAs, like miR-125a-3p, have been shown to increase sensitivity to chemotherapy and initiate apoptosis as well as senescence in ovarian cancer cells [85]. Emerging evidence supports that the combination of PARP inhibitors and compounds that modify miRNA expression or influence the DNA damage response pathways could be more effective in overcoming metastasis, highlighting the possibility of more complex treatment strategies for better results in patient care [86].

An integrated approach to ovarian cancer based on miRNA expression profiles and the use of PARP inhibitors results in greater understanding of their complementary and distinct actions. It, however, sheds light on new and exciting ways to enhance therapeutic efficacy and improve patient care (Table 3). The introduction of olaparib and subsequently, niraparib and rucaparib marked a new era in the treatment of ovarian cancer, especially for patients bearing BRCA mutations or confirmed HRD, as these drugs targeted the cancer using a neroplastic approach, taking advantage of the faulty DNA repair systems of the cancerous cells [87].

**Table 3:** Comparison of miRNA-based strategies and PARP inhibitor therapies in ovarian cancer

Parameter	miRNA-based therapies	PARP inhibitor therapies
Mechanism of action	Regulate multiple genes post-transcriptionally, including those involved in redox homeostasis, EMT, apoptosis, and DNA repair (e.g., SIRT1, PTEN, SESN2)	Inhibit PARP enzymes, preventing single-strand DNA repair, inducing synthetic lethality in BRCA-mutated cells
Key targets	SIRT1, MAPK14, PTEN, PI3K/AKT/mTOR, ZEB1, SESN2	PARP1/2, BRCA1/2-deficient DNA repair pathways
Patient selection	Based on redox status, miRNA expression profiles, treatment history	BRCA1/2 mutation or homologous recombination deficiency (HRD)
Biomarker utility	Circulating or exosomal miRNAs (e.g., miR-21, miR-29b, miR-200c) for diagnosis, prognosis, recurrence monitoring	BRCA1/2 mutation, HRD status
Delivery platform	Liposomes, exosomes, aptamer-functionalized nanoparticles, hydrogels	Oral capsules; approved dosing and pharmacokinetics
Clinical stage	Mostly preclinical or Phase I/II trials (e.g., MRX34, TargomiR)	FDA-approved (Olaparib, Rucaparib, Niraparib) for maintenance and BRCA-mutated advanced OC
Combination strategies	With chemotherapy, ferroptosis inducers, immune checkpoint inhibitors, or ROS modulators	With platinum-based chemotherapy or anti-angiogenic agents like bevacizumab
Advantages	High precision potential; target multiple hallmarks of cancer; non-invasive biomarkers; modulate redox state	Proven efficacy; oral administration; defined genetic targets
Limitations	Delivery efficiency, immune activation, off-target effects, regulatory standardization	Limited to HRD-positive tumors; hematologic toxicity, resistance development

*EMT* epithelial–mesenchymal transition; *OC* ovarian cancer; *HRD* homologous recombination deficiency; *PARP* poly (ADP-ribose) polymerase

While these therapies have now become standard in maintenance treatment, resistance—both intrinsic and acquired—remains a major clinical obstacle requiring the formulation of new treatment and biomarker innovations for patient stratification [88, 89].

Strategies involving miRNAs offer the possibility of targeting the regulatory systems controlling gene expression on a higher order, including those responsible for DNA repair, responses to oxidative stress, and drug resistance [90]. As biomarkers or therapeutics, miRNAs modulate critical pathways involved in tumor growth, metastasis, and resistance to chemotherapy. For instance, miRNA-221-5p can regulate the expression of DNA damage repair genes RAD18 and RAD51. Its restoration can sensitize chemoresistant ovarian cancer cells to platinum therapies, suggesting its valorization as a therapeutic target [39, 65, 72, 91].Further, some studies indicate that the therapeutic action of PARP inhibitors may be increased and resistance diminished when miRNA modulating PARP or other DNA damage response pathway regulators are used in combination.

As an illustrative example, the dual treatment of olaparib with ATR and CHK1 inhibitors not only enhances cytotoxicity and prevents migration and invasion of ovarian cancer cell lines, but also alters miRNAs associated with metastasis and drug resistance, indicating a possible synergistic effect [86]. Moreover, the Robelin [92] study showed that miR-125a-3p, which corroborates PARP inhibition, up-regulated after PARP inhibition exposing the interplay between PARP inhibition and miRNA cross-talk. Although this work is encouraging, the majority of miRNA therapies remain preclinical, with issues of delivery, specificity, and off-target effects. In contrast to PARP inhibitors, which are well-established clinically, the therapies are limited by the frequent resistance and the precise selection of the patients [93]. The combination of miRNA strategies with PARP inhibitors and other targeted therapies is an innovative approach that will reduce resistance and improve treatment results, needing cross-validation in large, biomarker-based clinical trials [94, 95]. Thus, we could conclude that, despite the innovation of PARP inhibitors in the treatment of ovarian cancer, the synergistic strategy of PARP inhibitors with miRNA-based therapies which are aimed to alter the multi-level controllers of malignancy and therapy resistance in the malignancy is very promising [96].

The potential ovarian cancer treatments of the future are designed to effectively combine both approaches to patient-specific selection, toxicity, and therapeutic impact and patient-specific selection, toxicity, and therapeutic impact [97]. Work is ongoing to optimize these factors. Some of these include the attempts to modify the regulatory miRNA networks of redox networks and resistance mechanisms in OC:

• Platinum resistant AKT pathway inhibition by anti-miR-214 is described in Yang et al. (2008).

• Li et al. (2020) describes miR-497 suppression is associated with reduced resistance to platinum agents through S1K1/mTOR pathway downregulation. These silencing miRNAs are involved in the circuits of resistance by intertwining with oxidative stress, handling of EMT sequentials, and DNA repair.

### Delivery systems for miRNA therapeutics

*Effective and targeted delivery* remains the cornerstone of miRNA therapy success. *Naked miRNAs degrade quickly and exhibit poor tissue retention*. Innovative delivery systems include:

- *Nanoparticles (e.g., liposomes, chitosan, PLGA)*: Improve stability and exploit the enhanced permeability and retention (EPR) effect for tumor targeting.
- *Ligand-functionalized nanoparticles* (e.g., folate, hyaluronic acid): Increase tumor specificity and bypass efflux transporters [98].
- *Exosome-based vectors*: Leverage natural cellular pathways and minimize immune recognition [47].
- *Hydrogels and aptamer–miRNA conjugates*: Allow for spatially controlled and sustained miRNA release.

*Co-delivery with chemotherapeutics or antioxidants can synergistically modulate the tumor microenvironment and overcome drug resistance*.

#### Challenges in clinical translation

The applciation of miRNA based strategies in the treatment of ovarian cancer still faces a myriad of obstacles clinically. Major concerns remain around the lack of specificity in targeting particular genes because the miRNAs can control multiple functions and genetic networks and biological interactions [70]. The delivery of miRNA mimics and inhibitors still remains a challenge. The transportation of these miRNA mimics and inhibitors to the tumor cells is still unresolved within the bounds of immunity and toxicity free systems.The ermergence of toxicity issues in clinical trials due to unestablished dosages raises safety concerns towards miRNA therapeutics [29]. The intricate miRNA networks around ovarian cancer, compounded by the lack of understanding of their roles, makes devising effective treatments exceedingly challenging [99]. Some evidence of immune mediated adverse events have also been reported in clinicals trials, The adverse effects that have been reported have not been effectively documented, therefore limiting the advancement of trials [70, 75]. There is growing optimism, however, in the development of biomedical engineering. Classifying patients through lesion spatial transcriptomics, programmable control through miRNA editing, and delivery through redox responsive systems are a few approaches that can drive precision medicine forward and reshape future clinical models [100].

## Consequences and future perspectives

MicroRNAs (miRNAs) are now recognized as important modulators of gene expression within redox-related pathways, which offers diagnostic and therapeutic potential in ovarian cancer [60]. Nevertheless, their clinical application is still limited by biological, technological, and regulatory factors— COVID complexities and guidelines—diverse molecular makeup and myriad of tumor types.

### Biological and clinical hurdles

The pleiotropic and context-dependent characteristics of microRNAs present formidable obstacles for clinical translation. Individual microRNAs frequently govern numerous mRNAs, producing extensive biological repertoires, which raises the risk of unintended off-target toxicity in healthy tissues [101]. Furthermore, their activity is modulated by microenvironmental gradients, the specific cancer subtype, and prevailing stress conditions,for instance, the miR-200 family can function as a suppressor under homeostatic conditions yet contribute to metastatic spread when oxidative stress is chronically elevated [102]. The separation of functionally relevant “driver” microRNAs from “passenger” bystanders remains a major analytical challenge, as overlapping, compensatory circuits among miRNA families mask the phenotypic consequences of individual family members. Compounding this, subtype-specific miRNA expression signatures in high-grade serous, endometrioid, and clear-cell ovarian carcinomas further constrain the reach of generic miRNA-directed therapies [103]. Moreover, the pervasive crosstalk among miRNAs, long non-coding RNAs, and circular RNAs introduces additional, poorly characterized layers of regulatory control.

### Technological innovations

Recent technical advances offer pathways to mitigate these complexities. Single-cell RNA sequencing and spatial transcriptomics now permit high-resolution quantification of redox-sensitive microRNAs within morphologically and functionally diverse tumor microregions [104]. Concurrently, artificial intelligence and machine-learning algorithms are being harnessed to model platinum resistance, delineate miRNA-mRNA regulatory circuits, and identify patients at elevated risk of aggressive disease progression [105]. Patient-derived organoids (PDOs constitute a three-dimensional in vitro model that faithfully recapitulates the genetic and phenotypic heterogeneity of the original tumor, thus enabling the dissection of microRNA-targeting strategies and the quantification of oxidative stress responses in a biologically relevant context,these characteristics render PDOs a powerful tool for the iterative refinement of personalized therapeutic regimens [106].

### Delivery and pharmacokinetic challenges

Despite technological advances, *miRNA pharmacokinetics and targeted delivery* remain major obstacles. *Unmodified miRNAs degrade rapidly in circulation and have limited cellular uptake* due to negative charge and size [107].

Nanotechnology-based platforms improve *stability, cellular uptake, and tumor specificity*. Examples include:

- *Lipid nanoparticles* for prolonged circulation
- *Polymeric carriers* for improved uptake under hypoxic conditions
- *Exosomes*, offering natural targeting and low immunogenicity [98]

Smart delivery systems that respond to *tumor pH, ROS levels, or temperature gradients* enable controlled payload release. Dual-function nanocarriers are also being developed to *simultaneously deliver miRNAs and scavenge ROS*.

**Fig. 4:**
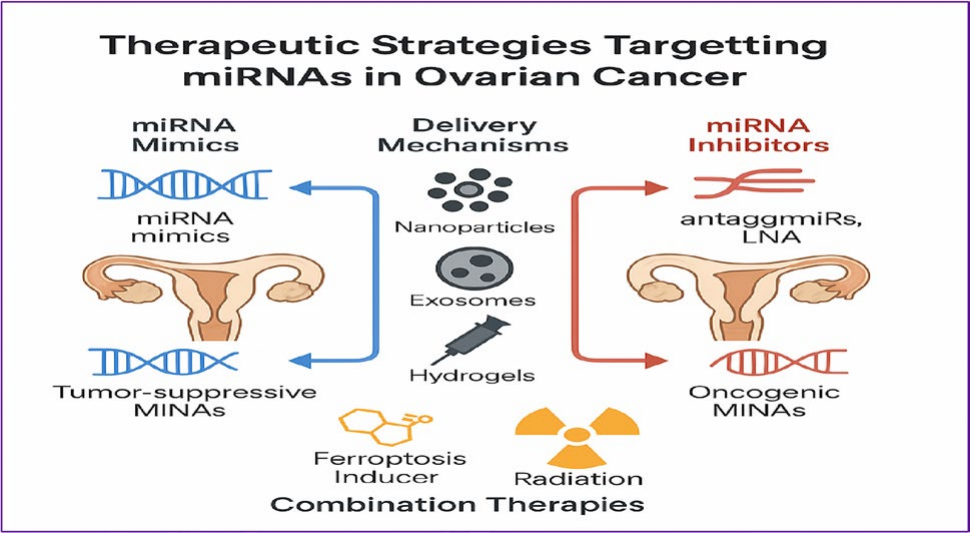
Therapeutic Strategies Targeting miRNAs in Ovarian Cancer. An integrative flowchart summarizing miRNA-based therapeutic approaches in OC, including miRNA mimics, antagomiRs, CRISPR/Cas-mediated editing, and ROS-responsive nanocarrier systems. The figure also outlines delivery platforms such as exosomes, hydrogels, and nanoparticles, along with their respective advantages and translational challenges

*However, limitations persist in terms of scalability, batch reproducibility, and targeting specificity for clinical use.* Figure 4 therapeutic strategies targeting miRNAs in ovarian cancer (see Table 4).

**Table 4:** Delivery platforms for miRNA therapeutics in ovarian cancer

Delivery system	Example	Advantages	Challenges
Nanoparticles	Chitosan NPs (miR-155-5p antagomiR)	Enhanced uptake; RNase protection	Potential toxicity; off-target effects
Exosomes	Tumor-derived vesicles	Natural targeting; low immunogenicity	Scalability; batch-to-batch variability
Hydrogels	Injectable, stimulus-responsive systems	Spatiotemporal control of release	Limited testing in ovarian cancer models
Aptamer–miRNA conjugates	EpCAM-targeted miRNA constructs	Specific delivery to tumor cells	Early-phase validation required

### CRISPR technology in miRNA therapeutics

*CRISPR/Cas systems offer high-precision tools* to edit miRNAs or their upstream regulators. For instance, *CRISPR/Cas9-mediated knockout of miR-21 suppresses proliferation and sensitizes tumors to chemotherapy* in OC models [15]. CRISPR/Cas13 allows direct editing of *mature miRNAs or untranslated regions*, enhancing control over non-coding RNA pathways [7]. *Nanocarrier-assisted CRISPR delivery systems* have shown improved tumor targeting and editing efficiency [108]. However, *standardized protocols for miRNA normalization and reproducibility* are still lacking [109], and *immune-related toxicity and dosing uncertainties* have limited clinical progress (e.g., MRX34, TargomiR) [63]. Nanocarrier and CRISPR-based miRNA therapeutics hold significant promise for advancing ovarian cancer treatment, yet their clinical translation is hampered by critical challenges such as toxicity, delivery efficiency, and specificity [15]. Nanocarriers, including DNA origami and various nanoparticle systems, can protect miRNA cargo from degradation and enhance tumor targeting, but issues remain regarding off-target effects, immunogenicity, and the potential for toxicity in healthy tissues [110]. The biological barriers and the diversity within tumor microenvironments make effective and accurate delivery to ovarian cancer cells the risk of side effects [111]. For CRISPR miRNA therapeutics, the primary concerns involve the delivery of gene-editing materials, off-target edits, and immune response [112, 113]. Improvements in targeting and decreases in toxicity are being worked on through the use of antibody-conjugated and extracellular vesicle-based delivery systems, but these methods are not ready for the clinic yet, and much work remains and optimization is needed [82, 112]. In any case, without first these problems being solved, the full therapeutic potential of nanocarrier and CRISPR miRNA on ovarian cancer therapies will remain unrealized [114].

### Future research directions

Future work should concentrate on the following to enable clinical translation:

•Targetable redox-responsive systems for miRNA delivery.

•Multicenter trials evaluating the diagnostic and prognostic value of specific miRNA panels.

•Redox phenotyping in combination with dynamic miRNA expression analysis to identify actionable environmental vulnerabilities.

The advancement of personalized medicine for ovarian cancer (OC) will be fueled by the integration of miRNA analytics, redox biology, and precision delivery systems.

### Validation challenges of microRNA‐based biomarkers

Validating the microRNA (miRNA) biomarkers in ovarian cancer with focus on oxidative stress, disease diagnosis, its mechanisms, and therapeutic resistance, poses some substantial hurdles. One of the main challenges is reproducibility across patient populations and techniques. Some studies indicate that while certain specific miRNA panels can accurately distinguish ovarian cancer cases from controls with high accuracy in the initial dataset, these panels often do not retain discriminatory accuracy in external validation cohorts. This is often due to the heterogeneity of the population, sample collection and processing methods that introduce confounding factors and limit the generalizability of the results [115, 116]. Other factors that complicate the validation of miRNA biomarkers are technical issues. The extraction and quantification of specific miRNAs, especially in exosomes or other extracellular vesicles, is very method-sensitive [117]. Fluctuations in techniques used to isolate exosomes will impact the purity and yield of miRNAs, resulting in differences in expression profiles and how well they serve as a diagnostic indicator. Standardized protocols for miRNA extraction and normalization, which are lacking, make cross-study comparisons impossible and limit the clinical translation of promising biomarkers [118]. Tumor biological variability, such as tumor and patient population heterogeneity, adds to these complications. Ovarian cancer is a subtype heterogeneous disease with multiple subtypes, each with its own distinct molecular and miRNA expression profiles [119]. Stage, subtype, and even the presence of other diseases can alter miRNA levels which complicates the ability to identify universal markers [120]. Therefore, to attain robust diagnostic accuracy, a diverse and large population is needed in order to validate panels of multiple miRNAs that are needed and ensure their clinical utility [121]. Integrating miRNA biomarkers with other diagnostic methods poses yet another challenge. The inclusion of miRNA panels alongside established protein markers such as CA-125 have proven to greatly enhance diagnostic accuracy, especially in early-stage ovarian cancer. But, to create joint models that include miRNA, protein, and clinical metadata, you need to use advanced statistics and machine learning, and these models need to be validated in other groups to make sure they work and can be reliably duplicated [122]. Ultimately, clinical translation of miRNA biomarkers is challenging due to the lack of primary large-scale, multi-center studies and multi-omic data integration.The vast amount of data generated from preclinical and clinical studies must be harmonized and analyzed using advanced bioinformatics tools [116]. Only through coordinated efforts to standardize methodologies, improve cohort diversity, and leverage integrative data analysis can reliable miRNA biomarkers be identified and validated for clinical use [118].

## Conclusion

The interplay between oxidative stress and dysregulated miRNAs is central to the initiation, progression, and therapeutic resistance of ovarian cancer. This review underscores how the reciprocal regulation between oxidative stress and miRNAs forms a feedback loop that modulates key oncogenic processes, including apoptosis evasion, epithelial–mesenchymal transition, angiogenesis, and chemoresistance. Redox-sensitive miRNAs, such as miR-29b, miR-200c, and miR-21, serve dual functions as both biomarkers and therapeutic targets, with their detectability in biofluids and tumor tissues supporting their application in non-invasive diagnostics and disease monitoring. We can now map miRNA-redox interactions with spatial transcriptomics, single-cell RNA sequencing, artificial intelligence modeling, and other emerging technologies. These innovations also improve the spatial and temporal precision with which miRNA-targeted therapies can be delivered. Although, issues like targeted delivery, drug metabolism, and how the body’s immune system would respond to the treatment, still makes it difficult to apply these methods in practice. The problem and treatment methods need to be combined. For instance, chemotherapy and immunotherapy can be combined with miRNA-based therapies to improve efficacy and treatment sustainability. We can use the miRNA regulation associated with oxidative stress to advance precision medicine in pediatric patients with long-term treatment resistance in advanced-stage ovarian cancer.

## Data Availability

No datasets were generated or analysed during the current study.

## References

[1] Abd El-Kader SM, Refay N, Atiaa AG. Coagulation, fibrinolytic parameters and cytokines response to weight reduction in obese Saudi women. J Med Rehabil Sci. 2025;2(1):21–8. 10.4197/Mrs.2-1.4.

[2] Saha S. Role of microrna in oxidative stress. Stresses. 2024;4(2):269–81. 10.3390/stresses4020016.

[3] Alharbi M, Sharma S, Guanzon D, Lai A, Zuñiga F, Shiddiky MJ, et al. MiRna signature in small extracellular vesicles and their association with platinum resistance and cancer recurrence in ovarian cancer. Nanomedicine Nanotechnol Biol Med. 2020;28:102207. 10.1016/j.nano.2020.102207. 10.1016/j.nano.2020.102207 32334098

[4] Luo Y, Liu X, Chen Y, Tang Q, He C, Ding X, et al. Targeting PAX8 sensitizes ovarian cancer cells to ferroptosis by inhibiting glutathione synthesis. Apoptosis. 2024;29(9–10):1499–514. 10.1007/s10495-024-01985-y. 38853202 10.1007/s10495-024-01985-y

[5] Aboelella NS, Brandle C, Kim T, Ding ZC, Zhou G. Oxidative stress in the tumor microenvironment and its relevance to cancer immunotherapy. Cancers. 2021;13(5):986. 10.3390/cancers13050986. 33673398 10.3390/cancers13050986 PMC7956301

[6] Ghantabpour T, Goudarzi N, Parsaei H. Overview of Nrf2 as a target in ovary and ovarian dysfunctions focusing on its antioxidant properties. J Ovarian Res. 2025;18:60. 10.1186/s13048-025-01639-w. 40121445 10.1186/s13048-025-01639-w PMC11929342

[7] Aloliqi AA, Alnuqaydan AM, Albutti A, Alharbi BF, Rahmani AH, Khan AA. Current updates regarding biogenesis, functions and dysregulation of microRNAs in cancer: innovative approaches for detection using CRISPR/Cas13-based platforms (review). Int J Mol Med. 2025;55(6):90. 10.3892/ijmm.2025.5531. 40242952 10.3892/ijmm.2025.5531 PMC12021393

[8] Mieszczański P, Januszyk S, Zmarzły N, Ossowski P, Dziobek K, Sagan D, et al. miRNAs participate in the regulation of oxidative stress-related gene expression in endometrioid endometrial cancer. Int J Mol Sci. 2022;23(24):15817. 10.3390/ijms232415817. 36555458 10.3390/ijms232415817 PMC9779631

[9] Fumimoto C, Yamauchi N, Minagawa E, Umeda M. MiR-146a is mutually regulated by high glucose-induced oxidative stress in human periodontal ligament cells. Int J Mol Sci. 2023;25(19):10702. 10.3390/ijms251910702. 10.3390/ijms251910702 PMC11476635 39409031

[10] Kartikasari AER, Michel-Lara P, Exton H, Tekin-Sari K, Alnefai EMM, Mitchell A, et al. Circulating micrornas as diagnostic biomarkers to detect specific stages of ovarian cancer: a comprehensive meta-analysis. Cancers. 2024;16(24):4190. 10.3390/cancers16244190. 39766088 10.3390/cancers16244190 PMC11674734

[11] Wang L, Liu LZ, Jiang BH. Dysregulation of micrornas in metal-induced angiogenesis and carcinogenesis. Semin Cancer Biol. 2021;76:279–86. 10.1016/j.semcancer.2021.08.009. 34428550 10.1016/j.semcancer.2021.08.009 PMC8627485

[12] Marí-Alexandre J, Carcelén AP, Agababyan C, Moreno-Manuel A, García-Oms J, Calabuig-Fariñas S, et al. Interplay between microRNAs and oxidative stress in ovarian conditions with a focus on ovarian cancer and endometriosis. Int J Mol Sci. 2019;20(21):5322. 10.3390/ijms20215322. 31731537 10.3390/ijms20215322 PMC6862266

[13] Mostafa MM, El-Aziz MKA, Ellakwa DES. MiRNA-mediated resistance mechanisms in prostate cancer: implications for targeted therapy and metastatic progression. Med Oncol. 2025;42(10):1–29. 10.1007/s12032-025-03006-7 40879856

[14] Sung H, Ferlay J, Siegel RL, Laversanne M, Soerjomataram I, Jemal A, et al. Global cancer statistics 2020: GLOBOCAN estimates of incidence and mortality worldwide for 36 cancers in 185 countries. CA Cancer J Clin. 2021;71(3):209–49. 10.3322/caac.21660. 33538338 10.3322/caac.21660

[15] Raza A, Fatima P, Yasmeen B, Rana ZA, Ellakwa DES. From resistance to remedy: the role of clustered regularly interspaced short palindromic repeats system in combating antimicrobial resistance—a review. Naunyn–Schmiedeberg’s Arch Pharmacol. 2024;397:1–15. 10.1007/s00210-024-02587-5. 10.1007/s00210-024-03509-6 39404843

[16] Neamah AS, Wadan AHS, Lafta FM, Elakwa DES. The potential role of targeting the leptin receptor as a treatment for breast cancer in the context of hyperleptinemia: a literature review. Naunyn-Schmiedebergs Arch Pharmacol. 2024. 10.1007/s00210-024-03592-9. 39565396 10.1007/s00210-024-03592-9

[17] Lu J, Zhen S, Li X. Characteristics of oxidative phosphorylation-related subtypes and construction of a prognostic signature in ovarian cancer. Curr Gene Ther. 2025;25(3):327–44. 10.2174/0115665232323373240905104033. 39289931 10.2174/0115665232323373240905104033

[18] Xu S, Fu GB, Tao Z, OuYang J, Kong F, Jiang BH, et al. MiR-497 decreases cisplatin resistance in ovarian cancer cells by targeting mTOR/P70S6K1. Oncotarget. 2015;6(28):26457–71. 10.18632/oncotarget.4762. 26238185 10.18632/oncotarget.4762 PMC4694914

[19] Raza A, Mushtaq N, Jabbar A, Ellakwa DES. Antimicrobial peptides: a promising solution to combat colistin and carbapenem resistance. Gene Rep. 2024;38:101935. 10.1016/j.genrep.2024.101935.

[20] Long Y, Shi H, Ye J, Qi X. Exploring strategies to prevent and treat ovarian cancer in terms of oxidative stress and antioxidants. Antioxidants. 2024;14(1):114. 10.3390/antiox14010114. 10.3390/antiox14010114 PMC11762571 39857448

[21] Ellakwa DES, Amr KS, Zaki ME, Refeat M, Banksle HM. Zinc finger 259 gene polymorphisms in Egyptian patients with metabolic syndrome and its association with dyslipidemia. Irish J Med Sci (1971–). 2024;193(5):2313–23. 10.1007/s11845-023-03467-0. 10.1007/s11845-024-03752-z 38985417

[22] Borhan WH, Atiaa AGF, Mohamed SEA, Ali KM. Response of immune system and wound healing to laser puncture in burned patients. Egypt J Hosp Med. 2023;90(1):590–4. 10.21608/EJHM.2023.260191.

[23] Atiaa AG, Abd E-Kader SM, Ellakwa DES. Integrative physiotherapy in burn rehabilitation: innovations in physical, psychological, and technological interventions–a narrative review. J Bodyw Mov Ther. 2025;45:74–82. 10.1016/j.jbmt.2025.07.023. 41316645 10.1016/j.jbmt.2025.07.023

[24] Khan Y, Rizvi S, Raza A, Khan A, Hussain S, Khan NU, et al. Tailored therapies for triple-negative breast cancer: current landscape and future perceptions. Naunyn-Schmiedebergs Arch Pharmacol. 2025. 10.1007/s00210-025-03896-4. 40029385 10.1007/s00210-025-03896-4

[25] Barrera G, Cucci MA, Grattarola M, Dianzani C, Muzio G, Pizzimenti S. Control of oxidative stress in cancer chemoresistance: spotlight on Nrf2 role. Antioxidants (Basel). 2021;10(4):510. 10.3390/antiox10040510.PMID:33805928;PMCID:PMC8064392. 33805928 10.3390/antiox10040510 PMC8064392

[26] Chen L, Wang K, Li L, Zheng B, Zhang Q, Zhang F, et al. Plasma exosomal miR-1260a, miR-7977 and miR-192-5p as diagnostic biomarkers in epithelial ovarian cancer. Future Oncol (London, England). 2022;18(26):2919–31. 10.2217/fon-2022-0321. 10.2217/fon-2022-0321 35893704

[27] Mirzaei S, Mohammadi AT, Gholami MH, Hashemi F, Zarrabi A, Zabolian A, et al. Nrf2 signaling pathway in cisplatin chemotherapy: potential involvement in organ protection and chemoresistance. Pharmacol Res. 2021;167:105575. 10.1016/j.phrs.2021.105575. 33771701 10.1016/j.phrs.2021.105575

[28] Xu Z, Yao T, Liu W. Mir-378a-3p sensitizes ovarian cancer cells to cisplatin through targeting MAPK1/GRB2. Biomed Pharmacother. 2018;107:1410–7. 10.1016/j.biopha.2018.08.132. 30257357 10.1016/j.biopha.2018.08.132

[29] Supruniuk E, Baczewska M, Żebrowska E, Maciejczyk M, Lauko KK, Milewska P, et al. Redox biomarkers and matrix remodeling molecules in ovarian cancer. Antioxidants. 2024;13(2):200. 10.3390/antiox13020200. 38397798 10.3390/antiox13020200 PMC10885995

[30] Yuan H, Hua Z, Zhang H, et al. Ferroptosis and ovarian cancer: a bibliometric study and visualization analysis. Discov Oncol. 2025;16:1572. 10.1007/s12672-025-03332-2. 40824457 10.1007/s12672-025-03332-2 PMC12361024

[31] Zhao H, Xu Y, Shang H. Ferroptosis: a new promising target for ovarian cancer therapy. Int J Med Sci. 2022;19(13):1847–55. 10.7150/ijms.76480. 36438923 10.7150/ijms.76480 PMC9682507

[32] Park MN, Kim M, Lee S, Kang S, Ahn CH, Tallei TE, et al. Targeting redox signaling through exosomal microrna: insights into tumor microenvironment and precision oncology. Antioxidants (Basel, Switzerland). 2025;14(5):501. 10.3390/antiox14050501. 40427384 10.3390/antiox14050501 PMC12108341

[33] Song C, Liu B, Xu P, Ge X, Li H, Tang Y, et al. MiR-144 is the epigenetic target for emodin to ameliorate oxidative stress induced by dietary oxidized fish oil via Nrf2 signaling in Wuchang bream, *Megalobrama amblycephala*. Aquaculture. 2021;534:736357. 10.1016/j.aquaculture.2021.736357.

[34] Meshkovska Y, Abramov A, Mahira S, Thatikonda S. Understanding the impact of oxidative stress on ovarian cancer: advances in diagnosis and treatment. Future Pharmacol. 2024;4(3):651–75. 10.3390/futurepharmacol4030035.

[35] Ellakwa DES, El Nakeeb SMS, Abd El Mohsen SA. The impact of hydrogen sulfide on mesenchymal stem cells in rats suffering from liver fibrosis via suppression of TGF-β signaling. Gene Rep. 2024;37:102056. 10.1016/j.genrep.2024.102056.

[36] Ellakwa DES, Elsheikh-Hassan AF, Ellakwa TE, Abdelmalek MA. Recent update on future therapeutic strategies for COVID-19 vaccination with Omicron variant. Hum Gene. 2024. 10.1016/j.humgen.2024.201281.

[37] Ebrahimi SO, Reiisi S, Shareef S. miRNAs, oxidative stress, and cancer: a comprehensive and updated review. J Cell Physiol. 2020;235(11):8812–25. 10.1002/jcp.29724. 32394436 10.1002/jcp.29724

[38] Abd El-Kader SM, AlKhateeb AM, AlFawaz SS, Neamatallah ZA, Alabasi UM, Gaowgzeh RM, et al. Balance deficit among diabetic polyneuropathy Saudi patients. J Med Rehabil Sci. 2025;2(1):8–13. 10.4197/Mrs.2-1.2.

[39] Ellakwa DES, Abdelmalek MA, Mostafa MM, Ellakwa TE, Wadan AHS. MircoRNAs predict and modulate responses to chemotherapy in leukemic patients. Naunyn-Schmiedebergs Arch Pharmacol. 2025. 10.1007/s00210-024-03675-7. 39808312 10.1007/s00210-024-03675-7

[40] Zhao J, Liu L, Zhao W, Lv C, Zhang N, Jia X, et al. Mir-141-3p accelerates ovarian cancer progression and promotes M2-like macrophage polarization by targeting the Keap1-Nrf2 pathway. Open Med. 2023;18(1):20230729. 10.1515/med-2023-0729. 10.1515/med-2023-0729 PMC10276613 37333452

[41] Li H, Wang X, Mu H, Mei Q, Liu Y, Min Z, et al. Mir-484 contributes to diminished ovarian reserve by regulating granulosa cell function via YAP1-mediated mitochondrial function and apoptosis. Int J Biol Sci. 2022;18(3):1008–21. 10.7150/ijbs.68028. 35173533 10.7150/ijbs.68028 PMC8771835

[42] Wang X, Yang J, Li H, Mu H, Zeng L, Cai S, et al. miR-484 mediates oxidative stress-induced ovarian dysfunction and promotes granulosa cell apoptosis via SESN2 downregulation. Redox Biol. 2023;62:102684. 10.1016/j.redox.2023.102684. 36963287 10.1016/j.redox.2023.102684 PMC10060268

[43] Xu J, Jia Y. MiR-361-5p regulates SLC25A24 to maintain mitochondrial function and alleviate granulosa cell dysfunction in diminished ovarian reserve. J Assist Reprod Genet. 2025;42(3):923–36. 10.1007/s10815-024-03349-6. 39810070 10.1007/s10815-024-03349-6 PMC11950524

[44] Chen SN, Chang R, Lin LT, Chern CU, Tsai HW, Wen ZH, et al. MicroRNA in ovarian cancer: biology, pathogenesis, and therapeutic opportunities. Int J Environ Res Public Health. 2019;16(9):1510. 10.3390/ijerph16091510. 31035447 10.3390/ijerph16091510 PMC6539609

[45] Fasoulakis Z, Psarommati MZ, Papapanagiotou A, Pergialiotis V, Koutras A, Douligeris A, et al. MicroRNAs can influence ovarian cancer progression by dysregulating integrin activity. Cancers (Basel). 2023;15(18):4449. 10.3390/cancers15184449. 37760437 10.3390/cancers15184449 PMC10526761

[46] Dahiya N, Sherman-Baust CA, Wang TL, Davidson B, Shih IeM, Zhang Y, et al. Microrna expression and identification of putative miRNA targets in ovarian cancer. PLoS ONE. 2008;3(6):e2436. 10.1371/journal.pone.0002436. 18560586 10.1371/journal.pone.0002436 PMC2410296

[47] Imran K, Iqbal MJ, Abid R, Ahmad MM, Calina D, Sharifi-Rad J, et al. Cellular signaling modulated by miRNA-3652 in ovarian cancer: unveiling mechanistic pathways for future therapeutic strategies. Cell Commun Signal. 2023;21(1):289. 10.1186/s12964-023-01330-x. 37845675 10.1186/s12964-023-01330-x PMC10577948

[48] Dari MAG, Jaberian Asl B, Dayer D, Azizidoost S, Farzaneh M, Salehi AM. MiR-34 as a critical regulator in ovarian cancer. Curr Mol Med. 2025. 10.2174/0115665240345216241120093846. 39773049 10.2174/0115665240345216241120093846

[49] Xu B, He T, Yang H, Dai W, Liu L, Ma X, et al. Activation of the p62-Keap1-Nrf2 pathway protects against oxidative stress and excessive autophagy in ovarian granulosa cells to attenuate DEHP-induced ovarian impairment in mice. Ecotoxicol Environ Saf. 2023;265:115534. 10.1016/j.ecoenv.2023.115534. 37776821 10.1016/j.ecoenv.2023.115534

[50] Fang G, Liu J, Wang Q, Huang X, Yang R, Pang Y, et al. RETRACTED: microRNA-223-3p regulates ovarian cancer cell proliferation and invasion by targeting SOX11 expression. Int J Mol Sci. 2017;18(6):1208. 10.3390/ijms18061208. 28587313 10.3390/ijms18061208 PMC5486031

[51] Feng P, Ge Z, Guo Z, Lin L, Yu Q. A comprehensive analysis of the downregulation of miRNA-1827 and its prognostic significance by targeting SPTBN2 and BCL2L1 in ovarian cancer. Front Mol Biosci. 2021;11(8):687576. 10.3389/fmolb.2021.687576.PMID:34179092;PMCID:PMC8226272. 10.3389/fmolb.2021.687576 PMC8226272 34179092

[52] Wang Y, Gao X, Wei F, Zhang X, Yu J, Zhao H, et al. Diagnostic and prognostic value of circulating miR-21 for cancer: a systematic review and meta-analysis. Gene. 2013;533(1):389–97. 10.1016/j.gene.2013.09.038. 24076132 10.1016/j.gene.2013.09.038

[53] Yoshida K, Yokoi A, Kato T, Ochiya T, Yamamoto Y. The clinical impact of intra- and extracellular miRNAs in ovarian cancer. Cancer Sci. 2020;111(10):3435–44. 10.1111/cas.14599. 32750177 10.1111/cas.14599 PMC7541008

[54] Wang S, Song X, Wang K, Zheng B, Lin Q, Yu M, et al. Plasma exosomal miR-320d, miR-4479, and miR-6763-5p as diagnostic biomarkers in epithelial ovarian cancer. Front Oncol. 2022;12:986343. 10.3389/fonc.2022.986343. 36591520 10.3389/fonc.2022.986343 PMC9795228

[55] Wang S, Wang C, Liu O, Hu Y, Li X, Lin B. MiRNA-651-3p regulates EMT in ovarian cancer cells by targeting ZNF703 and via the MEK/ERK pathway. Biochem Biophys Res Commun. 2022;619:76–83. 10.1016/j.bbrc.2022.06.005. 35749939 10.1016/j.bbrc.2022.06.005

[56] Ghafouri-Fard S, Shoorei H, Taheri M. Non-coding RNAs are involved in the response to oxidative stress. Biomed Pharmacother. 2020;127:110228. 10.1016/j.biopha.2020.110228. 32559852 10.1016/j.biopha.2020.110228

[57] Carbonell T, Gomes AV. MicroRNAs in the regulation of cellular redox status and its implications in myocardial ischemia-reperfusion injury. Redox Biol. 2020;36:101607. 10.1016/j.redox.2020.101607. 32593128 10.1016/j.redox.2020.101607 PMC7322687

[58] Xu C, Chen J, Tan M, Tan Q. The role of macrophage polarization in ovarian cancer: from molecular mechanism to therapeutic potentials. Front Immunol. 2025;16:1543096. 10.3389/fimmu.2025.1543096. 40330466 10.3389/fimmu.2025.1543096 PMC12052780

[59] Xu W, Guan G, Yue R, Dong Z, Lei L, Kang H, et al. Chemical design of magnetic nanomaterials for imaging and ferroptosis-based cancer therapy. Chem Rev. 2025;125(4):1897–961. 10.1021/acs.chemrev.4c00546. 39951340 10.1021/acs.chemrev.4c00546

[60] Putri HR, Novianti PW, Pradjatmo H, Haryana SM. Microrna-mediated approaches in ovarian cancer therapy: a comprehensive systematic review. Oncol Lett. 2024;28:491. 10.3892/ol.2024.14624. 39185494 10.3892/ol.2024.14624 PMC11342411

[61] Ellakwa TE, Ellakwa DE. Enhancement of the solubility and the dissolution rate of oral nimodipine formulation with solid dispersion. Egypt J Chem. 2021;64(2):721–8. 10.21608/ejchem.2020.43731.2782.

[62] Ellakwa DES, Mushtaq N, Khan S, Jabbar A, Abdelmalek MA, Wadan AHS, et al. Molecular functions of micrornas in colorectal cancer: recent roles in proliferation, angiogenesis, apoptosis, and chemoresistance. Naunyn-Schmiedebergs Arch Pharmacol. 2024. 10.1007/s00210-024-03076-w. 38619588 10.1007/s00210-024-03076-w

[63] Ellakwa DES, Abdel-Hamid M, Ashour MSED, Khairy LES, Ali OSM. Identifying of HBV DNA in liver tissues of chronic hepatitis and hepatocellular carcinoma to study the Hepatitis B virus silent infection in Egyptian patients. Ecol Genet Genom. 2021;18:100077. 10.1016/j.egg.2020.100077.

[64] Mvunta DH, Miyamoto T, Asaka R, Yamada Y, Ando H, Higuchi S, et al. Overexpression of SIRT1 is associated with poor outcomes in patients with ovarian carcinoma. Appl Immunohistochem Mol Morphol. 2017;25(6):415–21. 10.1097/PAI.0000000000000316. 26862948 10.1097/PAI.0000000000000316

[65] Ellakwa DES, Mansour AS, Gorgui R, Banksle HM. Cerebrolysin in post-TBI recovery: Pharmacology and clinical evidence. Gene Reports. 2025;38:102202. 10.1016/j.genrep.2025.102202.

[66] Abd El-Kader SM, Refay N, Atiaa AG, Qawagzah SM. Bone turnover markers response to aerobic versus resistance exercise among postmenopausal Saudi women. J Med Rehabil Sci. 2025;2(1):14–20. 10.4197/Mrs.2-1.3.

[67] Ravegnini G, De Iaco P, Gorini F, Dondi G, Klooster I, De Crescenzo E, et al. Role of circulating miRNAs in therapeutic response in epithelial ovarian cancer: a systematic revision. Biomedicines. 2021;9(10):1316. 10.3390/biomedicines9101316. 34680433 10.3390/biomedicines9101316 PMC8533254

[68] Papadaki C, Monastirioti A, Rounis K, Makrakis D, Kalbakis K, Nikolaou C, et al. Circulating micrornas regulating DNA damage response and responsiveness to cisplatin in the prognosis of patients with non-small cell lung cancer treated with first-line platinum chemotherapy. Cancers. 2020;12(5):1282. 10.3390/cancers12051282. 32438598 10.3390/cancers12051282 PMC7281609

[69] Stieg DC, Wang Y, Liu LZ, Jiang BH. ROS and miRNA dysregulation in ovarian cancer development, angiogenesis and therapeutic resistance. Int J Mol Sci. 2022;23(12):6702. 10.3390/ijms23126702. 35743145 10.3390/ijms23126702 PMC9223852

[70] Shaaban EM, Ellakwa DE, Elaraby NM, Amr KS, Mohamadin AM. The effect of insulin-loaded gold and carboxymethyl chitosan nanoparticles on gene expression of glucokinase and pyruvate kinase in rats with diabetes type 1. J Food Biochem. 2022;46(12):e14447. 10.1111/jfbc.14447. 36219732 10.1111/jfbc.14447

[71] Wu D, Zhang Y, Zhang L, Xia W, Cai B, Dong F, et al. Mechanism of microRNA-152-3p-mediated regulation of autophagy and sensitivity in Paclitaxel-resistant ovarian cancer cells. Onco Targets Ther. 2025;18:179–97. 10.2147/OTT.S485100. (**Feb 4;**). 39926373 10.2147/OTT.S485100 PMC11806707

[72] Ellakwa DES, Rashed LA, Ali OS, El-Sabbagh NA. A study to determine the effect of nano-selenium and thymoquinone on the Nrf2 gene expression in Alzheimer’s disease. Future Sci OA. 2025;11(1):2458434. 10.2144/fsoa-2024-0081. 39887156 10.1080/20565623.2025.2458434 PMC11792829

[73] Xie S, Su Y, Zhang J, Yin F, Liu X. Upregulation of miRNA-450b-5p targets ACTB to affect drug resistance and prognosis of ovarian cancer via the PI3K/Akt signaling pathway. Transl Cancer Res. 2024;13(9):4800–12. 10.21037/tcr-24-292. 39430863 10.21037/tcr-24-292 PMC11483453

[74] Teng Y, Zhang Y, Qu K, Yang X, Fu J, Chen W, et al. Microrna-29B (mir-29b) regulates the Warburg effect in ovarian cancer by targeting AKT2 and AKT3. Oncotarget. 2015;6(38):40799–814. 10.18632/oncotarget.5695. 26512921 10.18632/oncotarget.5695 PMC4747369

[75] Ali HM, Ellakwa DES, Elaraby NM, Zaher AM, Amr KS. Study the association of microRNA polymorphisms (miR-146a, miR-4513) with the risk of coronary heart diseases in Egyptian population. J Biochem Mol Toxicol. 2023;37(3):e23284. 10.1002/jbt.23284. 36541377 10.1002/jbt.23284

[76] Wadan AHS, Ahmed MA, Ahmed AH, Ellakwa DES, Elmoghazy NH, Gawish A. The interplay of mitochondrial dysfunction in oral diseases: recent updates in pathogenesis and therapeutic implications. Mitochondrion. 2024;80:101942. 10.1016/j.mito.2024.101942. 10.1016/j.mito.2024.101942 39111357

[77] Behranvand N, Nasri F, Zolfaghari Emameh R, Khani P, Hosseini A, Garssen J, et al. Chemotherapy: a double-edged sword in cancer treatment. Cancer Immunol Immunother CII. 2022;71(3):507–26. 10.1007/s00262-021-03013-3. 34355266 10.1007/s00262-021-03013-3 PMC10992618

[78] Kartikasari AE, Exton H, Alnefai EM, Mitchell A, Plebanski M. Circulating microRNAs as diagnostic biomarkers to detect specific stages of ovarian cancer: a comprehensive meta-analysis. Cancers. 2023;16(24):4190. 10.3390/cancers16244190. 10.3390/cancers16244190 PMC11674734 39766088

[79] Jia YZ, Liu J, Wang GQ, Song ZF. miR-484: a potential biomarker in health and disease. Front Oncol. 2022;9(12):830420. 10.3389/fonc.2022.830420.PMID:35356223;PMCID:PMC8959652. 10.3389/fonc.2022.830420 PMC8959652 35356223

[80] Maeda K, Sasaki H, Ueda S, Miyamoto S, Terada S, Konishi H, et al. Serum exosomal microRNA-34a as a potential biomarker in epithelial ovarian cancer. J Ovarian Res. 2020;13(1):47. 10.1186/s13048-020-00648-1. 32336272 10.1186/s13048-020-00648-1 PMC7184688

[81] Elsabbagh NA, Ellakwa DE. The protective effects of nano-selenium particles and thymoquinone against rats’ lipopolysaccharide-induced Alzheimer’s disease. Azhar Int J Pharm Med Sci. 2025;5(2):248–61.

[82] Staicu CE, Predescu DV, Rusu CM, Radu BM, Cretoiu D, Suciu N, et al. Role of micrornas as clinical cancer biomarkers for ovarian cancer: a short overview. Cells. 2020;9(1):169. 10.3390/cells9010169. 31936634 10.3390/cells9010169 PMC7016727

[83] Kobayashi H, Yoshimoto C, Matsubara S, Shigetomi H, Imanaka S. A comprehensive overview of recent developments on the mechanisms and pathways of ferroptosis in cancer: the potential implications for therapeutic strategies in ovarian cancer. Cancer Drug Resist. 2023;6(3):547–66. 10.20517/cdr.2023.49. 37842240 10.20517/cdr.2023.49 PMC10571061

[84] Nwokwu CD, Xiao AY, Harrison L, Nestorova GG. Identification of microrna-mRNA regulatory network associated with oxidative DNA damage in human astrocytes. ASN Neuro. 2022;14:17590914221101704. 10.1177/17590914221101704. 35570825 10.1177/17590914221101704 PMC9118907

[85] Wang Z, Pu T, Miao W, Gao Y, Gao J, Zhang X. Olaparib increases chemosensitivity by upregulating miR-125a-3p in ovarian cancer cells. Discov Oncol. 2025;16(1):291. 10.1007/s12672-025-02048-7. 40064834 10.1007/s12672-025-02048-7 PMC11893969

[86] Gralewska P, Biegała Ł, Gajek A, Szymczak-Pajor I, Marczak A, Śliwińska A, et al. Olaparib combined with DDR inhibitors effectively prevents EMT and affects miRNA regulation in TP53-mutated epithelial ovarian cancer cell lines. Int J Mol Sci. 2025;26(2):693. 10.3390/ijms26020693. 39859407 10.3390/ijms26020693 PMC11766100

[87] Zhan S, Yung MM, Siu MK, Jiao P, Ngan HY, Chan DW, et al. New insights into ferroptosis initiating therapies (FIT) by targeting the rewired lipid metabolism in ovarian cancer peritoneal metastases. Int J Mol Sci. 2021;23(23):15263. 10.3390/ijms232315263. 10.3390/ijms232315263 PMC9737695 36499591

[88] Jiang Y, Song L, Lin Y, et al. ROS-mediated SRMS activation confers platinum resistance in ovarian cancer. Oncogene. 2023;42:1672–84. 10.1038/s41388-023-02679-6. 37020040 10.1038/s41388-023-02679-6 PMC10231978

[89] Kapper C, Oppelt P, Arbeithuber B, Gyunesh AA, Vilusic I, Stelzl P, et al. Targeting ferroptosis in ovarian cancer: novel strategies to overcome chemotherapy resistance. Life Sci. 2024;349:122720. 10.1016/j.lfs.2024.122720. 38762066 10.1016/j.lfs.2024.122720

[90] Ellakwa TE, Amin TR. Study on the inhibition kinetics parameters of esterases enzymes from the red palm weevil larvae. Ecol Genet Genomics. 2022;22:100113. 10.1016/j.egg.2022.100113.

[91] Ellakwa TE, Ellakwa A, Abu-Khadra AS, Gomaa HM, El-Taib Heakal F, El-Sayed Ellakwa D. Reviewing and exploring boron oxide’s role in bioactive glasses: a synthesis of modeling and applications. J Australian Ceram Soc. 2025;61(2):719–32. 10.1007/s41779-024-00297-5.

[92] Shaaban EM, Amr KS, Mohamadin AM, Ellakwa DE. The effect of insulin loaded nanoparticles on immuno-reactivity of beta cells in rats with diabetes type 1. Azhar Int J Pharm Med Sci. 2023;3(1):96–104. 10.21608/aijpms.2022.136796.1144.

[93] Zeytün E, Altıntop MD, Sever B, Özdemir A, Ellakwa DE, Ocak Z, et al. A new series of antileukemic agents: design, synthesis, in vitro and in silico evaluation of thiazole-based ABL1 kinase inhibitors. Anti-Cancer Agents Med Chem. 2021;21(9):1099–109. 10.2174/1871520620666200824100408 32838725

[94] Jeon H, Seo SM, Kim TW, Ryu J, Kong H, Jang SH, et al. Circulating exosomal miR-1290 for diagnosis of epithelial ovarian cancer. Curr Issues Mol Biol. 2022;44(1):288–300. 10.3390/cimb44010021. 35723400 10.3390/cimb44010021 PMC8928998

[95] Riaz R, Ahmed I, Raza A, Khan Y, Ahsan U, Ellakwa DES. Response of different infection models in broiler chickens against supplemental organic acid—a review. Microb Pathog. 2025;188:107527. 10.1016/j.micpath.2025.107527 40185170

[96] Abd El Mohsen SA, Rashed LA, El Nakeeb S, Ellakwa DE. An inhibitory effect of bone marrow-mesenchymal stem cells and hydrogen sulphide on liver fibrosis via the transforming growth factor β signaling. Azhar Int J Pharm Med Sci. 2025;5(2):46–56.

[97] Helmy A, Ellakwa TE, El-BassyounI GT, et al. Influence of vanadium oxide on the structural, optical, mechanical and dielectric properties of cadmium borate glasses. Sci Rep. 2025;15:27430. 10.1038/s41598-025-09064-1. 40721717 10.1038/s41598-025-09064-1 PMC12304156

[98] Alberto L, Rodríguez AL, Sahare P, Pathak S, Banerjee A, Duttaroy AK, et al. Applications of nanotechnologies for miRNA-based cancer therapeutics: current advances and future perspectives. Front Bioeng Biotechnol. 2023;11:1208547. 10.3389/fbioe.2023.1208547. 37576994 10.3389/fbioe.2023.1208547 PMC10416113

[99] Bhowmik A, McCaffrey MS, Rooney Varga J. From climate endgame to climate long game. Proc Natl Acad Sci U S A. 2022;119(45):e2214975119. 10.1073/pnas.2214975119. 36322727 10.1073/pnas.2214975119 PMC9659379

[100] Younis NF, Rashed LA, El-mandoury AA, Ellakwa DE. Genetic factors in preeclamptic Egyptian women: relation with arginine vasopressin; a case-control study. Azhar Int J Pharm Med Sci. 2025;5(1):202–15. 10.21608/aijpms.2024.185070.1186.

[101] Fu Z, Wang L, Li S, Chen F, Au-Yeung KK, Shi C. MicroRNA as an important target for anticancer drug development. Front Pharmacol. 2021;12:736323. 10.3389/fphar.2021.736323. 34512363 10.3389/fphar.2021.736323 PMC8425594

[102] Toiyama Y, Takahashi M, Hur K, Nagasaka T, Tanaka K, Inoue Y, et al. Serum miR-21 as a diagnostic and prognostic biomarker in colorectal cancer. J Natl Cancer Inst. 2013;105(12):849–59. 10.1093/jnci/djt101. 23704278 10.1093/jnci/djt101 PMC3687369

[103] Ellakwa DES, Rashed LA, El-Mandoury AAA, Younis NF. Epigenetic alterations in preeclampsia: a focus on microRNA149 and tetrahydrofolate reductase gene polymorphisms in Egyptian women. Irish J Med Sci (1971–). 2024;5:46. 10.1007/s11845-024-03732-3. 10.1007/s11845-024-03732-3 38848035

[104] Wadan AHS, Shaaban AH, El-Sadek MZ, Mostafa SA, Moshref AS, El-Hussein A, et al. Mitochondrial-based therapies for neurodegenerative diseases: a review of the current literature. Naunyn––Schmiedeberg’s Arch Pharmacol. 2025;398:1–30. 10.1007/s00210-025-02620-y. 10.1007/s00210-025-04014-0 PMC12449425 40163151

[105] Torre LA, Trabert B, DeSantis CE, Miller KD, Samimi G, Runowicz CD, et al. Ovarian cancer statistics, 2018. CA Cancer J Clin. 2018;68(4):284–96. 10.3322/caac.21456. 29809280 10.3322/caac.21456 PMC6621554

[106] Atiaa AG, Al Balah OF, Mostafa KAH, Abas DN, Abd El Wahab HH. Effect of low level laser therapy on nerve conduction velocity in diabetic neuropathic patients: a randomized controlled trial. Annals Romanian Soc Cell Biol. 2021;25(6):9338–46.

[107] Martino MTD, Tagliaferri P, Tassone P. Microrna in cancer therapy: breakthroughs and challenges in early clinical applications. J Exp Clin Cancer Res. 2025;44:126. 10.1186/s13046-025-03391-x. 40259326 10.1186/s13046-025-03391-x PMC12010629

[108] García-Guede Á, Vera O, Ibáñez-de-Caceres I. When oxidative stress meets epigenetics: implications in cancer development. Antioxidants (Basel, Switzerland). 2020;9(6):468. 10.3390/antiox9060468. 32492865 10.3390/antiox9060468 PMC7346131

[109] Precazzini F, Detassis S, Imperatori AS, Denti MA, Campomenosi P. Measurements methods for the development of microRNA-based tests for cancer diagnosis. Int J Mol Sci. 2021;22(3):1176. 10.3390/ijms22031176. 33503982 10.3390/ijms22031176 PMC7865473

[110] Han L, Song T, Wang X, Luo Y, Gu C, Li X, et al. Mir-21 responsive nanocarrier targeting ovarian cancer cells. Comput Struct Biotechnol J. 2024;24:196–204. 10.1016/j.csbj.2024.02.021. 38495121 10.1016/j.csbj.2024.02.021 PMC10940798

[111] Lou Y, Wang Y, Lu J, Chen X. Microrna-targeted nanoparticle delivery systems for cancer therapy: current status and future prospects. Nanomedicine (Lond). 2025;20(10):1181–94. 10.1080/17435889.2025.2492542. 40231694 10.1080/17435889.2025.2492542 PMC12068351

[112] Hussen BM, Rasul MF, Abdullah SR, et al. Targeting miRNA by CRISPR/Cas in cancer: advantages and challenges. Military Med Res. 2023;10:32. 10.1186/s40779-023-00468-6. 10.1186/s40779-023-00468-6 PMC10351202 37460924

[113] Kim HK, Cheong H, Kim MY, Jin HE. Therapeutic targeting in ovarian cancer: nano-enhanced CRISPR/Cas9 gene editing and drug combination therapy. Int J Nanomed. 2025;20:3907–31. 10.2147/IJN.S507688. 10.2147/IJN.S507688 PMC11970428 40191042

[114] Godbole N, Quinn A, Carrion F, Pelosi E, Salomon C. Extracellular vesicles as a potential delivery platform for CRISPR-Cas based therapy in epithelial ovarian cancer. Semin Cancer Biol. 2023;96:64–81. 10.1016/j.semcancer.2023.10.002. 37820858 10.1016/j.semcancer.2023.10.002

[115] Aalami AH, Abdeahad H, Mesgari M. Circulating miR-21 as a potential biomarker in human digestive system carcinoma: a systematic review and diagnostic meta-analysis. Biomarkers. 2021;26(2):103–13. 10.1080/1354750X.2021.1875504. 33434077 10.1080/1354750X.2021.1875504

[116] Frisk NL, Sørensen AE, Pedersen OB, Dalgaard LT. Circulating microRNAs for early diagnosis of ovarian cancer: a systematic review and meta-analysis. Biomolecules. 2023;13(5):871. 10.3390/biom13050871. 37238740 10.3390/biom13050871 PMC10216356

[117] Atiaa AG, Al Balah OF, Mostafa KAH, Abas DN, Abd El Wahab HH. Efficacy of ultrasound therapy versus conventional therapy on reducing multiple components of neuropathic pain in diabetic neuropathic patients – comparative study. Fizjoterapia Polska. 2021;21(2):154–8.

[118] Bhadra M, Sachan M. An overview of challenges associated with exosomal miRNA isolation toward liquid biopsy-based ovarian cancer detection. Heliyon. 2024;10(9):e30328. 10.1016/j.heliyon.2024.e30328. 38707279 10.1016/j.heliyon.2024.e30328 PMC11068823

[119] Tossetta G, Fantone S, Montanari E, Marzioni D, Goteri G. Role of NRF2 in ovarian cancer. Antioxidants. 2022;11(4):663. 10.3390/antiox11040663. 35453348 10.3390/antiox11040663 PMC9027335

[120] Kozłowski M, Borzyszkowska D, Golara A, Lubikowski J. The role of microRNA in the prognosis and diagnosis of ovarian cancer. Int J Mol Sci. 2024;26(7):3413. 10.3390/ijms26073413. 10.3390/ijms26073413 PMC11989830 40244333

[121] Hamidi F, Gilani N, Arabi Belaghi R, Yaghoobi H, Babaei E, Sarbakhsh P, et al. Identifying potential circulating miRNA biomarkers for the diagnosis and prediction of ovarian cancer using machine-learning approach: application of Boruta. Front Digit Health. 2023;9(5):1187578. 10.3389/fdgth.2023.1187578.PMID:37621964;PMCID:PMC10445490. 10.3389/fdgth.2023.1187578 PMC10445490 37621964

[122] Akbari A, Majd HM, Rahnama R, Heshmati J, Morvaridzadeh M, Agah S, et al. Cross-talk between oxidative stress signaling and microRNA regulatory systems in carcinogenesis: focused on gastrointestinal cancers. Biomed Pharmacother. 2020;131:110729. 10.1016/j.biopha.2020.110729. 33152911 10.1016/j.biopha.2020.110729

[123] Ellakwa TE, Abu-Khadra AS, Ellakwa DES. Influence of physico-chemical properties of hydroxypropyl methylcellulose on quetiapine fumarate release from sustained release matrix tablets. BMC Chem. 2024;18(1):219. 10.1186/s13065-024-01311-2. 39511691 10.1186/s13065-024-01311-2 PMC11545565

[124] Li J, Hu YP, Liang XL, Liu MW. Sodium Houttuyniae attenuates ferroptosis by regulating TRAF6-c-Myc signaling pathways in lipopolysaccharide-induced acute lung injury (ALI). BMC Pharmacol Toxicol. 2024;25(1):63. 10.1186/s40360-024-00787-x.PMID:39243105;PMCID:PMC11380410. 39243105 10.1186/s40360-024-00787-x PMC11380410

[125] Liu J, Yoo J, Ho JY, et al. Plasma-derived exosomal miR-4732-5p is a promising noninvasive diagnostic biomarker for epithelial ovarian cancer. J Ovarian Res. 2021;14:59. 10.1186/s13048-021-00814-z. 33910598 10.1186/s13048-021-00814-z PMC8082916

[126] Liu Q, Yang X, Yin Y, Zhang H, Yin F, Guo P, et al. Identifying the role of oxidative stress-related genes as prognostic biomarkers and predicting the response of immunotherapy and chemotherapy in ovarian cancer. Oxid Med Cell Longev. 2022;2022:6575534. 10.1155/2022/6575534. 36561981 10.1155/2022/6575534 PMC9764017

[127] Lu C, Zhou D, Wang Q, Liu W, Yu F, Wu F, et al. Crosstalk of microRNAs and oxidative stress in the pathogenesis of cancer. Oxid Med Cell Longev. 2020;28(2020):2415324. 10.1155/2020/2415324.PMID:32411322;PMCID:PMC7204110. 10.1155/2020/2415324 PMC7204110 32411322

[128] Meng F, Brandão M, Cullen JM. Replacing plastics with alternatives is worse for greenhouse gas emissions in most cases. Environ Sci Technol. 2024;58(6):2716–27. 10.1021/acs.est.3c05191. 38291786 10.1021/acs.est.3c05191 PMC10867844

[129] O’Neill CP, Dwyer RM. Nanoparticle-based delivery of tumor suppressor microRNA for cancer therapy. Cells. 2020;9(2):521. 10.3390/cells9020521. 32102476 10.3390/cells9020521 PMC7072816

[130] Pal R, Choudhury T, Ghosh M, Vernakar M, Nath P, Nasare VD. A signature of circulating miRNAs predicts the prognosis and therapeutic outcome of taxane/platinum regimen in advanced ovarian carcinoma patients. Clin Transl Oncol. 2024;26(7):1716–24. 10.1007/s12094-024-03394-8. 38472557 10.1007/s12094-024-03394-8

[131] Qadir MMF, Klein D, Álvarez-Cubela S, Domínguez-Bendala J, Pastori RL. The role of MicroRNAs in diabetes-related oxidative stress. Int J Mol Sci. 2019;20(21):5423. 10.3390/ijms20215423. 31683538 10.3390/ijms20215423 PMC6862492

[132] Reda El Sayed S, Cristante J, Guyon L, Denis J, Chabre O, Cherradi N. MicroRNA therapeutics in cancer: current advances and challenges. Cancers. 2021;13(11):2680. 10.3390/cancers13112680. 34072348 10.3390/cancers13112680 PMC8198729

[133] Robelin P, Tod M, Colomban O, Lachuer J, Ray-Coquard I, Rauglaudre G, et al. Comparative analysis of predictive values of the kinetics of 11 circulating miRNAs and of CA125 in ovarian cancer during first line treatment (a GINECO study). Gynecol Oncol. 2020;159(1):256–63. 10.1016/j.ygyno.2020.07.021. 32712155 10.1016/j.ygyno.2020.07.021

[134] Ruan D, Wen J, Fang F, Lei Y, Zhao Z, Miao Y. Ferroptosis in epithelial ovarian cancer: a burgeoning target with extraordinary therapeutic potential. Cell Death Discov. 2023;9(1):1–10. 10.1038/s41420-023-01721-6. 38040696 10.1038/s41420-023-01721-6 PMC10692128

[135] Selmy AM, Rashed LA, Ellakwa DE. Association between Kisspeptin and Foxa2 in regulation of trophoblast function and placentation: possible role in pregnancy viability. Azhar Int J Pharm Med Sci. 2025;5(1):118–27. 10.21608/aijpms.2024.268303.1255.

[136] Waris G, Ahsan H. Reactive oxygen species: role in the development of cancer and various chronic conditions. J Carcinog. 2006;11(5):14. 10.1186/1477-3163-5-14.PMID:16689993;PMCID:PMC1479806. 10.1186/1477-3163-5-14 PMC1479806 16689993

[137] Wei L, He Y, Bi S, Li X, Zhang J, Zhang S. miRNA-199b-3p suppresses growth and progression of ovarian cancer via the CHK1/E-cadherin/EMT signaling pathway by targeting ZEB1. Oncol Rep. 2021;45(2):569–81. 10.3892/or.2020.7895. 33416170 10.3892/or.2020.7895 PMC7757082

[138] Zhang F, Luo BH, Wu QH, Li QL, Yang KD. LncRNA HCG18 upregulates TRAF4/TRAF5 to facilitate proliferation, migration and EMT of epithelial ovarian cancer by targeting miR-29a/b. Mol Med. 2022;28(1):2. 10.1186/s10020-021-00415-y. 34983361 10.1186/s10020-021-00415-y PMC8725507

[139] Zhang W, Su X, Li S, Liu Z, Wang Q, Zeng H. Low serum exosomal miR-484 expression predicts unfavorable prognosis in ovarian cancer. Cancer Biomark. 2020;27(4):485–91. 32065786 10.3233/CBM-191123 PMC12662311

